# Pressure response of decylammonium-containing 2D iodide perovskites

**DOI:** 10.1016/j.isci.2022.104057

**Published:** 2022-03-11

**Authors:** Marta Morana, Rossella Chiara, Boby Joseph, Thomas B. Shiell, Timothy A. Strobel, Mauro Coduri, Gianluca Accorsi, Ausonio Tuissi, Angelica Simbula, Federico Pitzalis, Andrea Mura, Giovanni Bongiovanni, Lorenzo Malavasi

**Affiliations:** 1 Department of Chemistry and INSTM, Viale Taramelli 16, 27100 Pavia, Italy; 2 Earth and Planets Laboratory, Carnegie Institution for Science, Washington, DC 20015, USA; 3 Elettra-Sincrotrone Trieste S. C. P. A., S.S. 14 km 163.5 in Area Science Park, Basovizza, Trieste, Italy; 4 CNR Nanotec, Institute of Nanotechnology, c/o Campus Ecotekne, via Monteroni, 73100 Lecce, Italy; 5 CNR–Consiglio Nazionale Delle Ricerche, Istituto di Chimica Della Materia Condensata e di Tecnologie per L’Energia, Via Previati 1/e, 23900 Lecco, Italy; 6 Department of Physics, University of Cagliari, Cittadella Universitaria S.P. Monserrato-Sestu km 0.7, 09042 Monserrato, Italy

**Keywords:** Engineering, Materials chemistry, Materials characterization, Materials physics

## Abstract

Manipulation by external pressure of the optical response of 2D Metal Halide Perovskites (MHPs) is a fascinating route to tune their properties and promote the emergence of novel features. We investigate here DA_2_PbI_4_ and DA_2_GeI_4_ (DA = decylammonium) perovskites in the pressure range up to ∼12 GPa by X-ray powder diffraction, absorption, and photoluminescence spectroscopy. Although the two systems share a similar structural evolution with pressure, the optical properties are rather different and influenced by Pb or Ge. DA_2_PbI_4_ shows a progressive red shift from 2.28 eV (*P* = 0 GPa) to 1.64 eV at 11.5 GPa, with a narrow PL emission, whereas DA_2_GeI_4_, changes from a non-PL system at ambient pressure to a clear broadband emitter centered around 730 nm with an intensity maximum at about 3.7 GPa. These results unveil the role of the central atom on the nature of emission under pressure in 2D MHPs containing a long alkyl chain.

## Highlights

• Phase separation of 2D perovskites containing decylammonium as a function of pressure
• Strong red-shift of DA_2_PbI_4_ has a function of pressure from 2.28 to 1.64 eV
• Emergence of broad emission in DA_2_GeI_4_ by increasing pressure

## Graphical abstract

**Figure:**
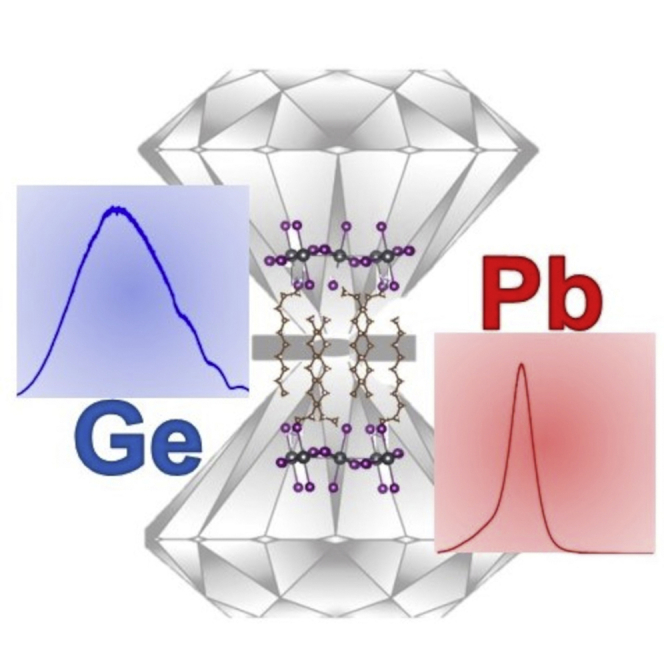

## Introduction

The exploration of the effect of external pressure application on metal halide perovskites (MHPs) is a topic of continuous interest because of the relevant and in some cases impressive modulation of the optical and electronic properties that can be induced on the MHP soft lattice at relatively low-pressure regimes (Jaffe et al., 2017; Li et al., 2019; Postorino and Malavasi, 2017; Shi et al., 2020; Szafrański and Katrusiak, 2017). The rich set of experimental and computational data as a function of pressure collected on 3D perovskites of general formula ABX_3_ (A= methylammonium, formamidinium, cesium, etc…; B=Pb, Sn, and Ge; X= Cl, Br, and I) allowed to highlight and define several common trends in the pressure-response of, for example, band gap and carrier lifetime, providing a solid basis to anticipate and predict the phase stability and electronic properties changes in these phases (Coduri et al., 2019, 2020; Jaffe et al., 2017; Li et al., 2019; Morana and Malavasi, 2021; Postorino and Malavasi, 2017; Seo et al., 1998; Shi et al., 2020; Szafrański and Katrusiak, 2017). In addition to the fundamental research interest of pressure-induced phenomena, the information collected *in situ* during pressure application may be possibly used at ambient conditions in devices by a proper modulation of stress/strain phenomena (Jiao et al., 2021; Zhu et al., 2019).

The study of pressure effects on the electronic and optical properties of so-called low-dimensional perovskites (LDP) and perovskite-derivatives is very limited, notwithstanding the current huge interest related to the vast possibility provided by the variation of the structural dimensions (2D, 1D, and 0D) and octahedral connectivity (corner-sharing, edge-sharing, and face-sharing) in this class of materials (Han et al., 2021; Li et al., 2021; Saidaminov et al., 2017; Zhou et al., 2019). Among the different types of LDPs, the 2D or layered systems of the Ruddlesden-Popper (RP) type are a hot topic of research due their high application potential in various fields ranging from photovoltaics, optoelectronic to photodetection (Gao et al., 2021; Li et al., 2021; Saidaminov et al., 2017; Wang et al., 2021; Zhang et al., 2019a). The available data about the pressure response of LDPs has been recently reviewed by Zhang et al. (Zhang et al., 2020). Differently from 3D perovskites, where the variation of the bond lengths and bond angles of the inorganic framework on pressure application plays the key role in the modulation of properties, the presence of a bulky organic component, layered among the inorganic layers in 2D perovskites, provides a further degree of structural complexity and may provide novel routes for the tuning of functional properties. The number of 2D MHPs prepared and investigated to date, particularly lead-based materials, is impressive. These studies, which include the rational design of the organic cation and halide, have shed light on the structure-property correlation in these systems providing a solid route to design tailored materials (Li et al., 2021).

On the other hand, the number of high-pressure studies of 2D MHPs of the RP series, with *n* = 1, i.e.*,* compounds of general formula A_2_BX_4_, is very limited and only focused on a few types of organic spacers, even though these specific LDP are among the structural series of major interest for applications (Zhang et al., 2020). (BA)_2_PbI_4_ (BA = butylammonium) has been object of intense High Pressure (HP) studies by various authors since 2004 (Matsuishi et al., 2004; Yin et al., 2019; Yuan et al., 2019). A thorough investigation by Yuan et al. up to 40 GPa indicates the presence of three phase transitions at 0.22, 2.2 and 13.1 GPa, with the coexistence of phase II and III in the range 2–7 GPa; however, it should stressed that the sample shows a clear on-set of amorphization already from about 4 GPa (Yuan et al., 2019). The band gap of (BA)_2_PbI_4_, determined from absorbance measurements, first presents a blue-shift at about 0.22 GPa from 2.28 to 2.37 eV, followed by a progressive band gap narrowing up to 0.95 eV at around 35 GPa (Yuan et al., 2019). The trend of photoluminescence (PL) data confirms the behavior of absorbance measurements with a peak in the PL intensity around 2 GPa, which eventually vanishes around 6–7 GPa. Both PL and absorbance data point toward the presence of a wide two-phase region below 10 GPa, as evidenced by HP X-ray diffraction (XRD) data. An analogous 2D perovskite with a longer alkyl chain (octylammonium) has been investigated up to 25 GPa in 2001 (Matsuishi et al., 2001). The authors reported the trend of PL and absorbance only, without any structural evidence as a function of *P*. A progressive reduction of the band gap from about 2.4 to 1.9 eV is observed in the *P* range from ambient pressure to about 12 GPa (Matsuishi et al., 2001). From the reported data it is possible to observe the presence of multiple absorption edges and PL peaks (up to three) from about 2 GPa to about 8 GPa which are interpreted by the authors as originating from bound-excition states. Other phenomena such as the disappearance of the excitonic state are discussed in terms of a change in the band structure from direct to indirect. Alternatively, as can be indirectly inferred from the reported plots of the optical properties, the observed trends could be due to the presence of a system composed by more than one structural phase as occurs in the (BA)_2_PbI_4_. It is interesting to note that in these (C_*n*_H_2*n*+1_NH_3_)_2_PbI_4_ perovskites, for *n* = 4 and 8, the trend of the band gap is different with respect to lead-based 3D perovskites where, after a red shift at relatively low pressures, a progressive blue-shift occurs, usually accompanied by structural amorphization (Postorino and Malavasi, 2017). The presence of the organic spacer, while not directly influencing the electronic structure, can play a templating role by affecting the inorganic sublattice, which indirectly modulates the optoelectronic properties in metal halide perovskites. In this respect it is of key importance to try to understand, in a systematic way, the role of the organic spacer on the properties of 2D MHPs. For example, the structural and optical studies carried out on the (C_*n*_H_2*n*+1_NH_3_)_2_PbI_4_ perovskites as a function of the number of carbon atoms (*n*) of the alkylammonium cation, helped researchers to rationalize the role of chain length on the properties of 2D perovskites such as electronic structure, exciton binding energies, orientation and melting of the organic chain, phase transitions, etc. (Billing and Lemmerer, 2007, 2008; Lemmerer and Billing, 2012; Sichert et al., 2019).

Triggered by the actual interest on LDPs and by the limited evidence of a relevant pressure-response in these systems, we conducted an HP study of the (C_10_H_21_NH_3_)_2_PbI_4_ 2D perovskite, i.e. containing the decylammonium (DA) cation, by XRD, PL and absorbance measurements as a function of pressure. Moreover, because the metal central atom plays a crucial role on the electronic structure of MHPs, we were interested in comparing the HP effects for a similar system but containing a different metal, namely germanium. We recently started to investigate the structure-property correlation in Ge-based 2D perovskites with the aim of understanding how the replacement of this ion for lead affects the structural, electronic, and optical properties, also considering the significant environmental problem raised by lead use and the necessity of finding possible lead-free alternatives (Malavasi et al., 2021a, 2021b). To the best of our knowledge, the present paper not only provides the first high-pressure experimental work on DA_2_PbI_4_ and DA_2_GeI_4_, the latter being a novel perovskite, but also represents the only available work of the pressure response of a Ge-based 2D MHPs.

## Results and discussion

### High pressure XRD and optical characterization of DA_2_PbI_4_

**Figure 1:**
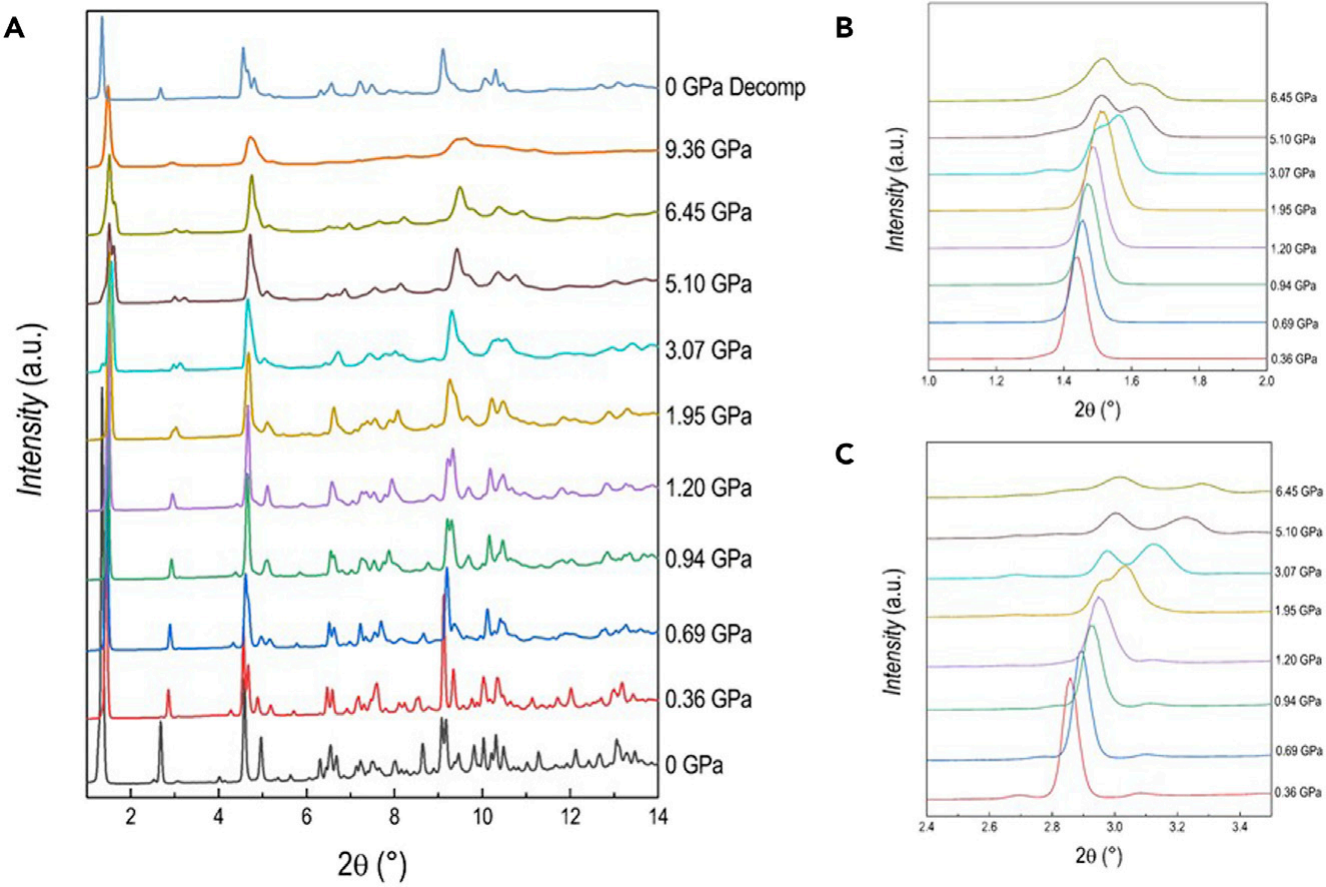
Diffraction data of DA_2_PbI_4_ (A–C) (A) XRD patterns (λ = 0.495 Å) as a function of pressure (reported in GPa on the right) for DA_2_PbI_4_; (B) and (C) highlights selected intervals of the pattern to better shown the evolution of the (001) and (002) reflections as a function of pressure.

DA_2_PbI_4_ has been prepared by wet-chemistry route, as described in the Experimental Section, and the ambient temperature and pressure XRD pattern can be refined in the orthorhombic *Pbca* space group as reported previously (see Figure S1) (Lemmerer and Billing, 2012). High-pressure XRD data have been collected at the XPress beamline, Elettra synchrotron, from ambient pressure to 9.4 GPa by using silicone oil as pressure transmitting medium (PTM) at the wavelength of 0.495 Å (further details are provided in the SI). An overview of the diffraction patterns collected as a function of the applied pressure is reported in Figure 1, also including the pattern after pressure release (“0 GPa decomp” at the top of Figure 1).

**Figure 2:**
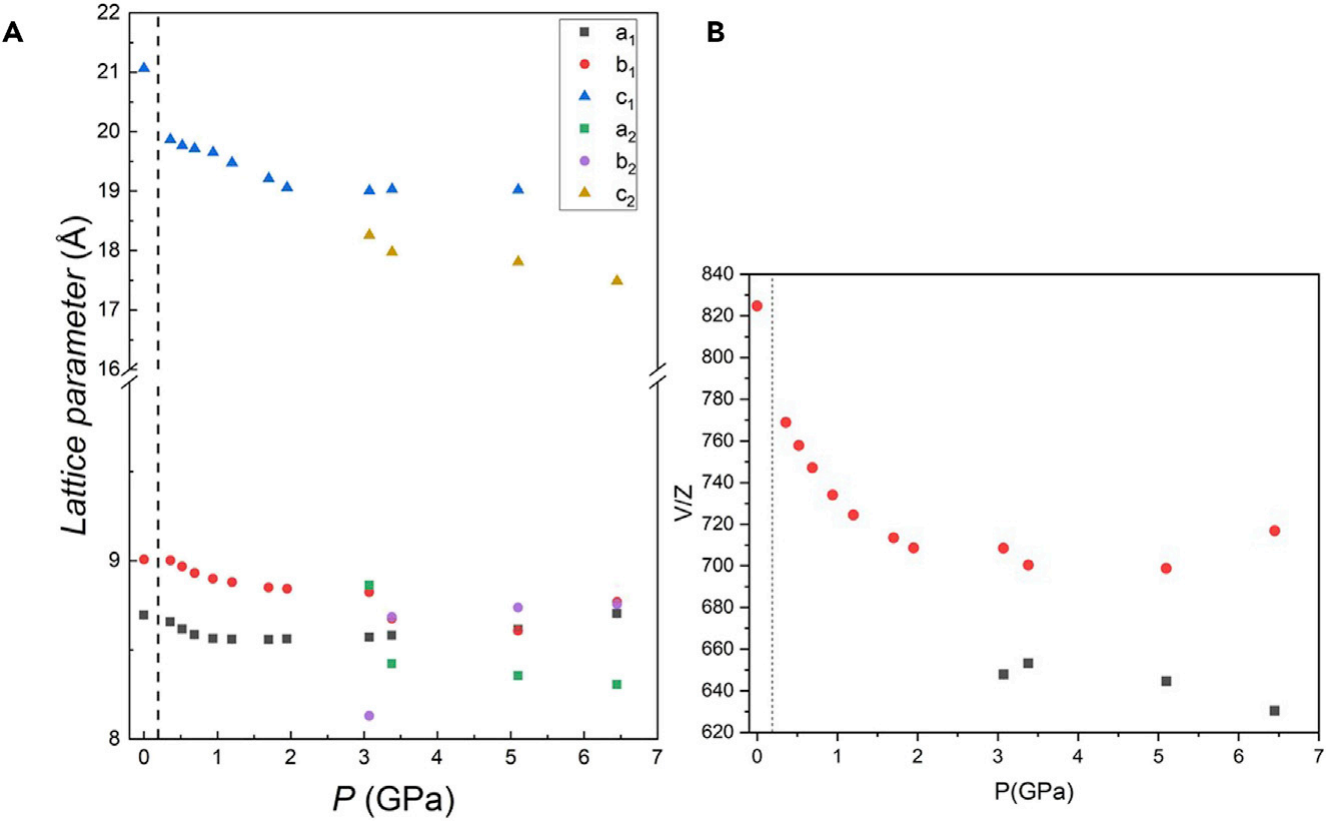
Pressure dependence of structural parameters of DA_2_PbI_4_ (A and B) Pressure dependence of the cell parameters a, b, c (A) and of the cell volume (B) for DA_2_PbI_4_. The c axis of the orthorhombic cell was halved. The dashed line marks the transition from the orthorhombic to the monoclinic cell. Subscripts 1 and 2 refer to the two monoclinic phases. Error bars are smaller than the symbol.

A sketch of ambient pressure DA_2_PbI_4_ structure together with bond angles and lengths of the inorganic framework are reported in Figure S2. Already at the first pressure investigated, namely 0.36 GPa, the crystal structure can no longer be indexed to the ambient conditions orthorhombic *Pbca* cell, and a monoclinic cell in the space group *P*2_1_/*a* has been found to properly describe the crystal structure. This symmetry is very close to the one obtained from single crystal data at low temperature (243 K) (Lemmerer and Billing, 2012). A similar transition has been reported also for the (BA)_2_PbI_4_ at ≈ 2 GPa, again in analogy with the phase transition sequence as a function of temperature (Billing and Lemmerer, 2007; Yuan et al., 2019). The refinement of the data at 0.36 GPa was then performed in the space group *P*2_1_/*a*, obtained from single crystal data (see Figure S3) (Lemmerer and Billing, 2012). According to the structural study by Lemmerer and Billing, this *Pbca*→*P*2_1_/*a* transformation is mostly due to changes in the inorganic layers that assume an eclipsed arrangement of adjacent layers, while the unit cell axis halves perpendicular to the layers (Lemmerer and Billing, 2012). The DA_2_PbI_4_ sample retains the same symmetry up to 1.70 GPa, when the peaks at approximately 1.5 and 3°, corresponding to the single (001) and (002) reflections in the *P*2_1_/*a* symmetry, start to show a splitting that increases upon compression (Figures 1B and 1C). This phenomenon clearly indicates the presence of two distinct phases in the sample and the patterns were now refined with two phases with space group *P*2_1_/*a* characterized by the *c*-axes differing approximately 2 Å each other. Notably, the intensity of the (001) reflection initially decreases till 3.07 GPa, then it increases again on further compression indicating a variation of the relative phase amount with pressure. In this respect, considering the past literature data and the results of the present manuscript, it seems that a common trend of (C_*n*_H_2*n*+1_NH_3_)_2_PbI_4_ perovskites is the evolution, upon the application of pressure, to multiple-phase systems (Lemmerer and Billing, 2012; Matsuishi et al., 2001). At 6.45 GPa the broadening of the peaks increases, and this effect appears more significant at 9.36 GPa, suggesting the onset of partial amorphization. The evolution of the cell parameters and lattice volume is summarized in Figures 2A and 2B.

After pressure release, the pattern can be indexed again with the orthorhombic cell, suggesting at least a certain degree of reversibility of the transition (Figure S4). However, the long-range structural integrity of the sample has been reduced after pressure application, but the position of the most intense peaks in the pattern (00*l*) can be still found at the same positions as the starting sample (Figure S4).

**Figure 3:**
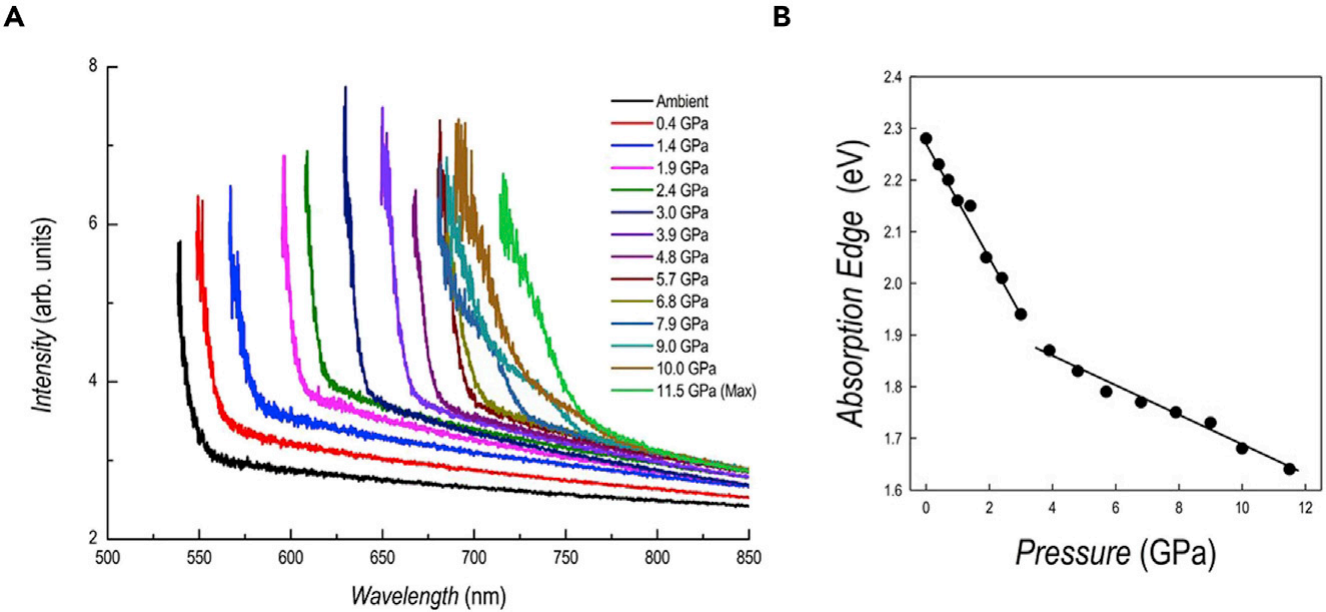
Absorption spectroscopy results of DA_2_PbI_4_ (A and B) (A) absorption spectra as a function of pressure for DA_2_PbI_4_; (B) trend of the absorption edge extracted from the spectra as a function of pressure.

**Figure 4:**
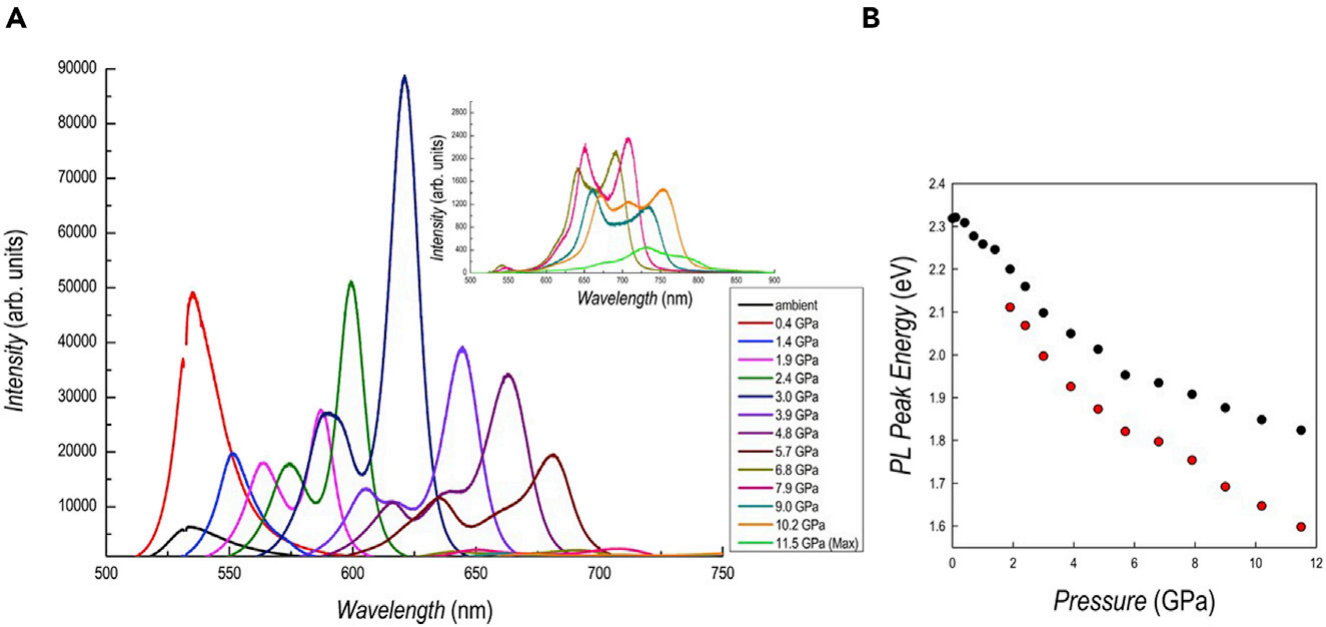
Photoluminescence results of DA_2_PbI_4_ (A and B) (A) PL spectra as a function of pressure for DA_2_PbI_4_; (B) trend of the peak position of the PL data as a function of pressure.

The optical properties as a function of pressure have been investigated by absorption and PL spectroscopies and the data are reported in Figures 3A and 4A, respectively.

The absorption data show a progressive and continuous red shift by increasing pressure. In general, the spectra reveal sharp edges even in the two-phase region determined by XRD, differently from the PL results (see below). A significant broadening of the spectra, with the appearance of a shoulder, is evident only in the spectra at 7.9 and 9 GPa. The trend of the absorption onset energy determined from the Tauc plot of the spectra of Figure 3A are reported in Figure 3B. The energy progressively decreases from the ambient pressure value of 2.28 eV, in agreement with previous literature data, down to 1.64 eV at 11.5 GPa (Sichert et al., 2019). No sign of variation is found in the region corresponding to the *Pbca*→*P*2_1_/*a* phase transition (0–0.36 GPa), while a different trend is observed (see continuous lines in Figure 3B) around the region where a two-phase system is found according to diffraction. The trend of absorption during decompression is fully reversible with the spectra at ambient pressure and after pressure release being superimposable (Figure S5).

The PL spectra as a function of pressure are shown in Figure 4A while Figure S6 shows optical micrographs of the sample inside the cell. A single PL peak is found up to 1.4 GPa (blue curve in Figure 4A) while from 1.9 GPa two distinct peaks are clearly visible in the spectra, with the second one shifted at higher wavelengths. This phenomenon is most probably related to the presence, starting from 1.9 GPa, of two distinct monoclinic phases in the sample, as shown by XRD analysis, with PL being more sensitive than absorbance in detecting this phase separation effect. The evolution of the peak position, expressed in eV, as a function of pressure is plotted in Figure 4B.

The intensity of the first peak (lower wavelength) increases passing from ambient pressure to 0.4 GPa (*Pbca*→*P*2_1_/*a* transition) and then gradually reduces its intensity. On the other hand, the low-energy peak intensity progressively increases up to 3 GPa and then decreases up to 5.7 GPa. The PL intensity of both peaks suddenly drops at 6.8 GPa even though the peaks continue to shift at lower energy (see the inset of Figure 4A). The trend of the first PL peak (black dots in Figure 4B) as a function of pressure is very similar to the trend of the band gap extracted from the absorbance data, with a slope deviation around 2 GPa. Possibly, the second peak could be ascribed to the second monoclinic phase with a more compressed *c*-axes characterized by a low energy band gap (red dots in Figure 4B). Similarly to the absorbance data, the PL after pressure release is superimposable to the one at ambient pressure, confirming the full reversibility of the sample (cf. Figure S7).

The trend of the absorbance and PL results may be interpreted considering the progressive Pb-I bond length shortening induced by pressure which gradually narrows the band gap, as commonly found in 3D perovskites (Postorino and Malavasi, 2017). The presence of two distinct PL peaks is related to the presence of two different phases in the sample from about 2 GPa. As can be seen, the two PL peak maxima are well separated and the one at a higher wavelength has a more pronounced red shift with respect to the peak at lower wavelength. As we demonstrated with *in situ* XRD data, the two monoclinic phases have a difference in the *c*-axis of about 2 Å. It is reasonable to ascribe the low-energy PL peak to the more compressed phase, also considering the trend of the *c*-axes of both phases shown in Figure 2A, which in some way reconciles with the behavior of the PL maxima of the two peaks. Some of the results reported here for DA_2_PbI_4_ have been observed also in BA_2_PbI_4_ (BA = butylammonium), namely the coexistence of two phases, the trend of the peak positions and intensity. A marked difference between the two samples is related to the absence of any blue-shift at low-pressure. The presence of a blue shift is common in 3D perovskites and is related to the increase deviation from 180° of Pb-I-Pb bond angle, widening the band gap (Postorino and Malavasi, 2017). For a short alkyl chain, namely four carbon atoms, such an effect is still present whereas in DA_2_PbI_4_, having a longer chain, only a progressive red shift has been observed. Moreover, it is interesting to note that such red shift is present passing from the orthorhombic to the monoclinic phase and is continuous in such a crystal symmetry. In general, the more distorted octahedra of the monoclinic phase leads to an increase of the band gap, which is not the present case. It is highly probable that the origin of this effect is related to the variation of the organic spacer conformation which progressively becomes more distorted while leading to less tilted and more compressed PbI_6_ octahedra and therefore band gap narrowing. This hypothesis has been formulated for the BA_2_PbI_4_ perovskite where, by increasing pressure, even in a more distorted phase, the octahedra result to be uncorrugated but, due to the low scattering contribution of the organic spacer, it is hard to gain solid insight into the orientation of the organic molecules (Yuan et al., 2019). A conclusion which may be drawn from the reported data is that a longer organic chain in lead-based 2D perovskites allows to reduce the tilting effects induced by pressure application on the inorganic framework (intralayer compression), making the layer-to-layer compression dominant and as the source of the observed continuous red shift. To corroborate this observation it is possible to mention that by increasing the number of inorganic layers in the (BA)_2_(MA)_n−1_Pb_n_I_3n+1_ system, i.e., making the 2D progressively “more 3D”, the pressure range of the red shift and the value at which the band gap blue-shift occurs progressively lowers (Liu et al., 2018).

### High pressure XRD and optical characterization of DA_2_GeI_4_

An analogous experimental approach has been used to investigate the HP behavior of DA_2_GeI_4_, which presents as an orange powder. DA_2_GeI_4_ is a novel material, and its crystal structure has never been determined before. A sketch of the crystal structure together with bond angles and lengths of DA_2_GeI_4_ is reported in Figure S8. In addition, as mentioned above, this is the first study of the pressure response of any Ge-based 2D perovskite. Indexing and refinement of powder diffraction data at ambient pressure suggest that the compound crystallize in the orthorhombic system (*Pbca*), as the lead analogue DA_2_PbI_4_ (Lemmerer and Billing, 2012). The refined pattern at ambient pressure is reported in Figure S9.

**Figure 5:**
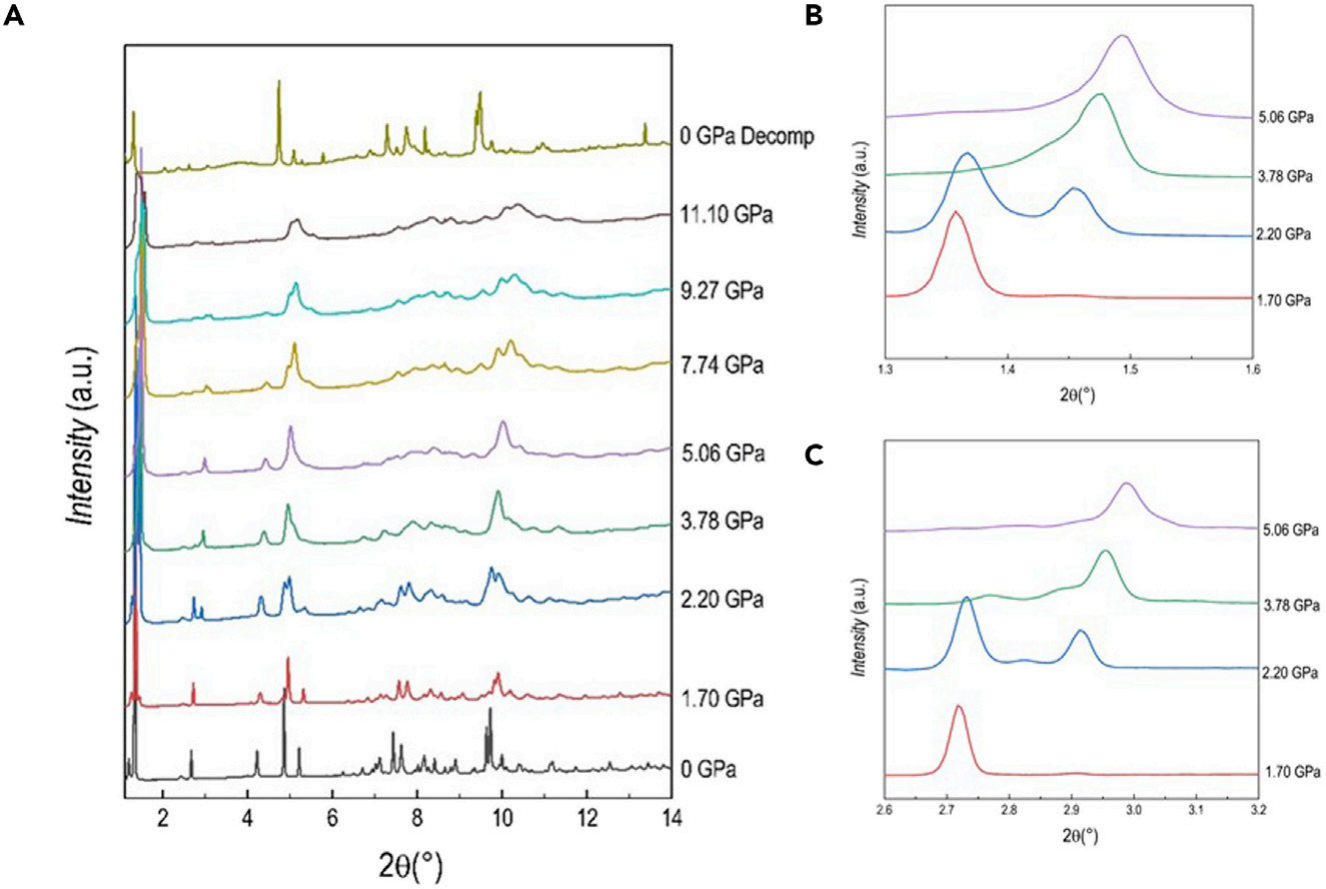
Diffraction data of DA_2_GeI_4_ (A–C) (A) XRD patterns (λ = 0.495 Å) as a function of pressure (reported in GPa on the right) for DA_2_GeI_4_; (B) and (C) highlights selected intervals of the pattern to better show the evolution of the (001) and (002) reflections as a function of pressure.

HP XRD data have been collected up to 11.1 GPa every ∼0.5 GPa and some selected patterns are reported in Figure 5A.

**Figure 6:**
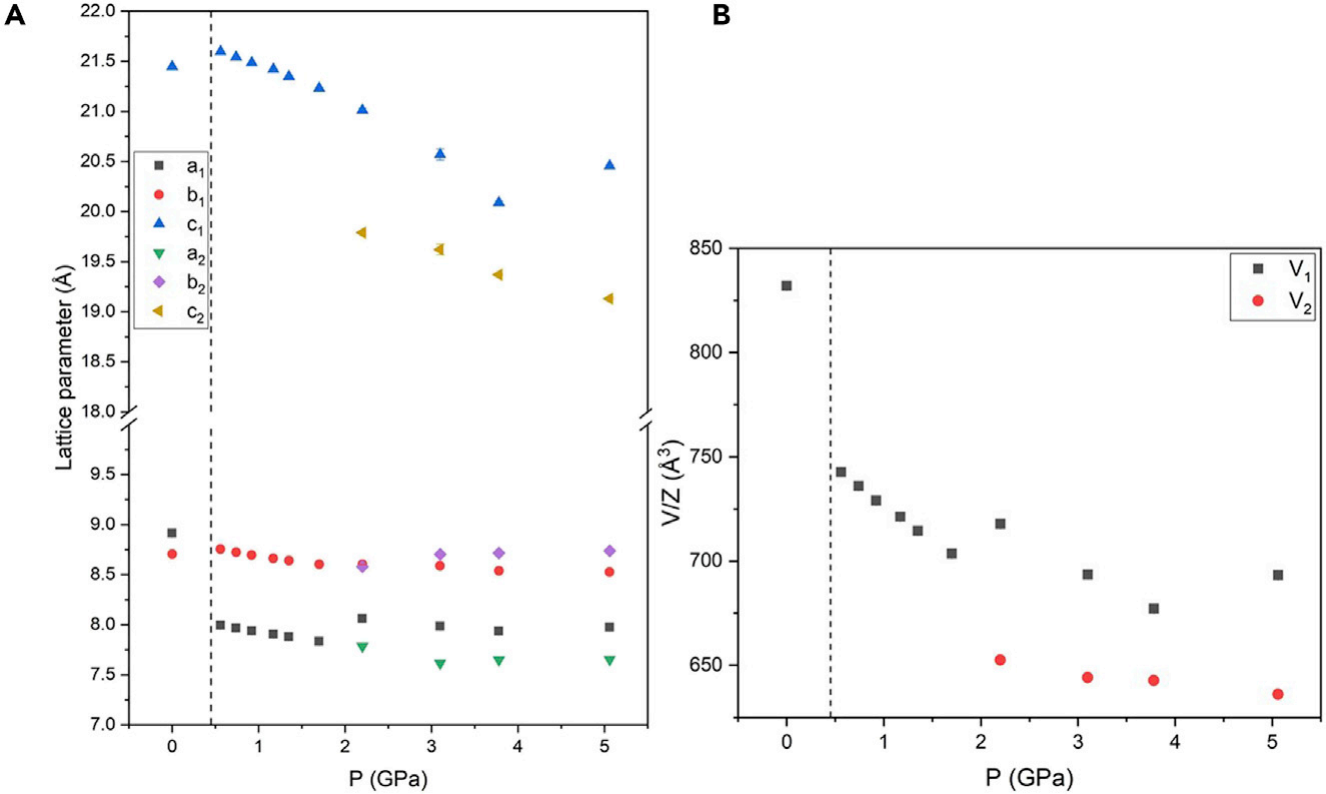
Pressure dependence of structural parameters of DA_2_GeI_4_ (A and B) Pressure dependence of the cell parameters a, b, c (A) and of the cell volume (B) for DA_2_GeI_4_. The c axis of the orthorhombic cell was halved. The dashed line marks the transition from the orthorhombic to the monoclinic cell. Error bars are smaller than the symbol.

The first high pressure pattern at 0.56 GPa shows a monoclinic symmetry in analogy with DA_2_PbI_4_ (see refined pattern as Figure S10). The sample persists as a single phase up to 2.20 GPa, when a splitting of the peaks at approximately 1.4 and 2.8° is clearly detectable (Figures 5A and 5B). Beyond this pressure, the patterns were refined with two phases with space group *P*2_1_/*a*, where the *c* axes differ by approximately 1 Å. In particular, the intensity of the (001) reflection of the first phase decreases while the (001) reflection of the second phase increases (Figure 5B) as a function of pressure. Already around 3.8 GPa, the phase with longer *c*-axes represents a significantly minority phase. A similar behavior occurs for the (002) reflections around 2.8° (Figure 5C). The two phases clearly coexist up to 5.06 GPa when the broadening of the peaks increases. The trend of lattice parameters and lattice volume as a function of pressure are reported in Figures 6A and 6B, respectively.

As mentioned above, DA_2_GeI_4_ is a novel compound and to get some insight into the structural behavior observed under pressure we collected variable-temperature laboratory XRD patterns. The most interesting result has been observed by heating the sample from room temperature (RT). Data are reported in Figure S11. At 50°C we could observe a phase separation of the sample, analogous to what has been observed by applying pressure. In general, such an effect corresponds to a disordering or even melting of the organic spacer (Billing and Lemmerer, 2007, 2008; Lemmerer and Billing, 2012). Further heating to 80°C leads to the restoration of a single phase (at least with the detection limits of our measurements). The common coexistence of two structural phases by the disordering/melting of the organic spacer by applying pressure and by increasing temperature is an interesting result which merits a further detailed investigation. As a matter of fact, it is known that in (C_*n*_H_2*n*+1_NH_3_)_2_PbI_4_ perovskites there is a complex and rich phase transition behavior due not only to the inorganic framework but also to the ordering of the organic spacer (Billing and Lemmerer, 2007, 2008; Lemmerer and Billing, 2012).

**Figure 7:**
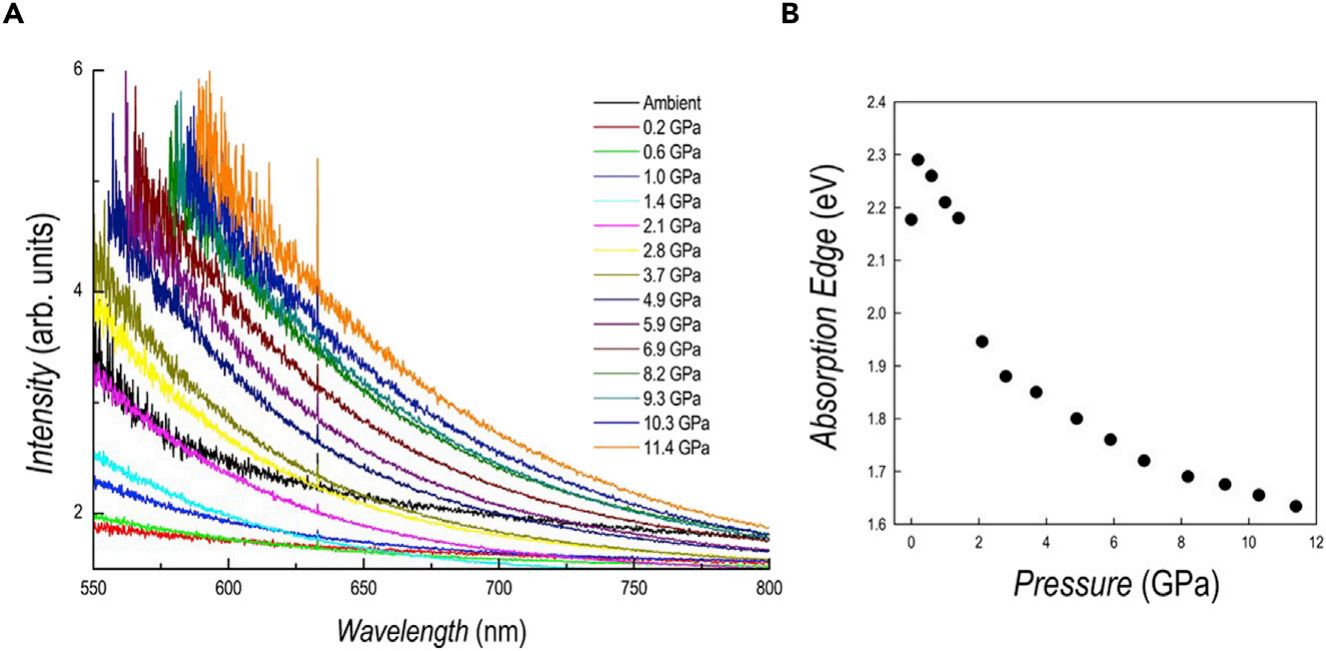
Absorption spectroscopy results of DA_2_GeI_4_ (A and B) (A) absorption spectra as a function of pressure for DA_2_GeI_4_. The peak at 633 nm is from the HeNe calibration laser; (B) trend of the absorption edge extracted from the spectra as a function of pressure.

**Figure 8:**
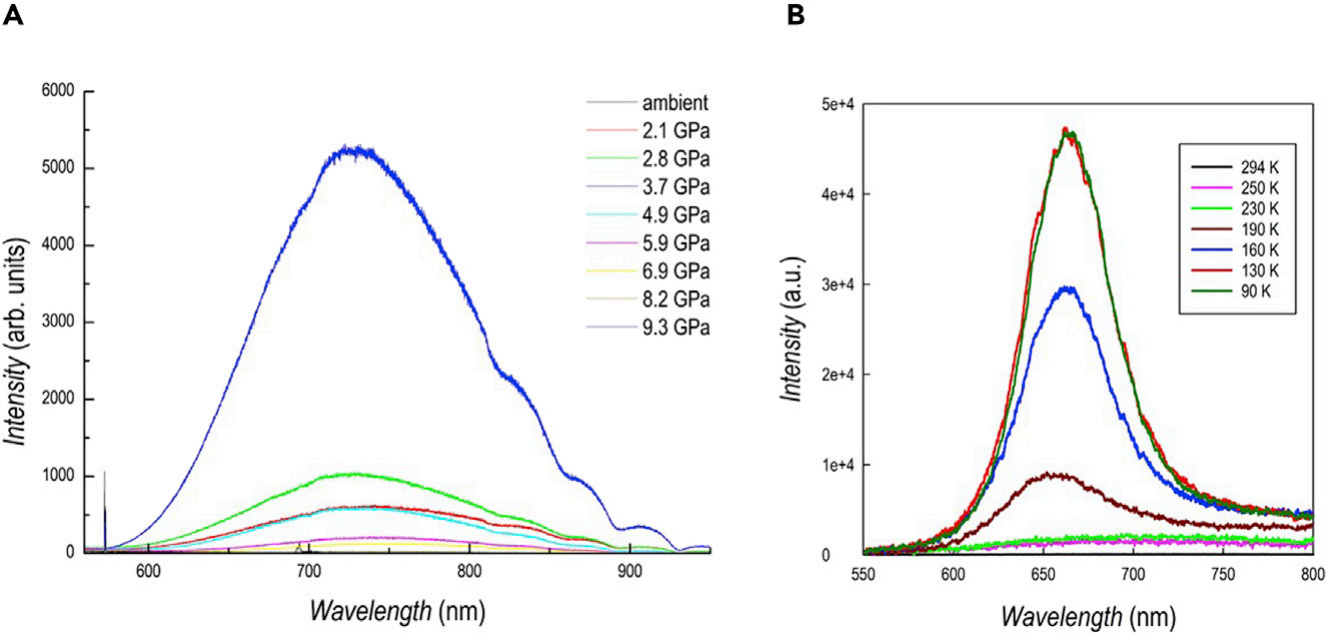
Photoluminescence results of DA_2_GeI_4_ (A and B) (A) PL data under compression for DA_2_GeI_4_. Oscillations above ∼800 nm are caused by detector etaloning; (B) PL data as a function of temperature for DA_2_GeI_4._

The optical properties as a function of pressure have been studied by absorption (Figure 7A) and PL (Figure 8B) spectroscopy.

The spectra as a function of pressure for DA_2_GeI_4_ show a sudden blue shift after the application of a moderate pressure (0.2 GPa) which may correspond to the set-up of the orthorhombic to monoclinic transition, differently from DA_2_PbI_4_. Further pressure application gradually red shift the absorbance and, at 2.1 GPa, the spectra is nearly superimposable onto that collected at ambient pressure (cf. Figure 7A). The red shift is then continuous until the maximum pressure investigated (11.4 GPa). With respect to DA_2_PbI_4_, the absorption edges are less defined and this is a feature quite common in lead-free perovskites with a possible origin in their reduced defect-tolerance (Xu et al., 2021). The values of the absorption onset extracted from the Tauc plots (except for some points at low pressure where, given the broad nature of the transitions, we used the maximum absorbance approach) show a change of slope and a sudden decrease in correspondence of about 2 GPa, i.e., the pressure where the patterns were refined with two phases with space group *P*2_1_/*a* and the *c* axes that differ by approximately 1 Å (see above the XRD section). As we pointed out earlier, the phase with shorter *c* axis is dominant around 3 GPa, and this reconciles with the absence of multiple absorption edge in the spectra of Figure 7A.

The trend of PL data for DA_2_GeI_4_ is reported in Figure 8A. Figure S12 reports optical micrographs of the sample inside the diamond anvil cell (DAC) during compression. At ambient pressure the sample has no detectable PL at room temperature while, from 2.1 GPa, a very broad peak, centered around 740 nm (1.67 eV) and with a Stokes shift >70 nm, emerges and reaches it maximum intensity around 3.7 GPa. The Full width at half maximum (FWHM) of the peak is ∼170 nm and both peak position and FWHM have a very modest dependence with pressure. Such behavior is fully reversible during decompression (cf. Figure S13) and, for comparison, the peak position at 3.7 GPa is 737 nm (compression), and 732 nm at 4.4 GPa (decompression), and the FWHM are 156 and 152 nm, respectively.

The evidence of a broadband emission by DA_2_GeI_4_ under pressure is a remarkable and interesting result. It is known that broadband emission in 2D perovskites is closely related to structural distortion induced by the organic spacers and by a wise chemical selection it is possible to modulate such an effect (Cortecchia et al., 2017; Smith et al., 2017). In addition, we recently demonstrated that Ge-based 2D perovskite have an intrinsic higher degree of octahedra distortion in the bond-length distribution affecting the optical properties (Malavasi et al., 2021a). The emergence of broadband distortion around 2.1 GPa may be also correlated to the emergence of the novel monoclinic phase with reduced *c*-axes (majority phase) which nicely couples to the trend of the band gap shown in Figure 7B. This first evidence of broadband emission by a 2D Ge-containing perovskite under moderate pressure is important to design suitable lead-free materials with analogous properties by playing with the chemical nature of the organic spacer at ambient pressure. The only previous indication, at ambient pressure, of broad emission in a 2D perovskite with Ge was due to Mitzi, and minimal data on pressure-induced broadband emission in 2D perovskite (all lead-based and containing bromide) have been reported to date, namely on PEA_2_PbBr_4_, BA_2_PbBr_4_, and (4BrPhMA)_2_PbBr_4_ (Gómez et al., 2020; Mitzi, 1996; Shen et al., 2020; Zhang et al., 2019b). In these cases, the origin of broad emission was found in self-trapped excitions (STEs) generated by lattice structural distortions, but it can also originate from permanent materials defects (Smith and Karunadasa, 2018). The pressure-induced emergence of wide emission in DA_2_GeI_4_, after the transition from the orthorhombic to the monoclinic phase, may suggest that an increase of structural distortion plays a role but, with the available datasets, we are not in the position of unequivocally assign the observed broad emission to self-trapping phenomena. A detailed discussion about the relaxation mechanisms in 2D perovskites can be found in Smith and Karunadasa (2018) (Smith and Karunadasa, 2018). The different behavior in the two perovskites investigated here, namely DA_2_PbI_4_ (narrow emission) and DA_2_GeI_4_ (broad emission) having a different central atom, but showing similar structural features at ambient and under applied pressure, is most likely correlated to the more significant distortion induced by germanium on octahedral bond elongation and bond angle variance, and less on the Ge–Br–Ge bond angle, with respect to Pb-containing perovskite (Malavasi et al., 2021a). This also couples to the dependence of the broadband emission with the increase of out-of-plane distortion in similar lead-based 2D perovskites (Malavasi et al., 2021a). It is also known that native (or structural related) defects may contribute to self-trapping phenomena related to broad PL, and it is possible that this phenomenon may contribute to the observed high-pressure optical properties evolution of DA_2_GeI_4_ because, in general, lead-free materials are less defect tolerant (Cortecchia et al., 2017). The possible origin of broad emission in DA_2_GeI_4_ was further investigated by low-temperature PL measurements (Figure 8B). While still being a non-PL emitter at room temperature, a broad PL signal centered around 665 nm (with a quantum yield around 17% at 77 K, see Figure S14) starts to be clear around 190 K and its intensity progressively increases by reducing temperature up to 130 K. The broad emission at low temperature has a smaller Stokes shift with respect to the one induced by pressure, being peaked around 665 nm, possibly because of the different extent of the lattice distortions induced by temperature and pressure. As a matter of fact, during pressure application, DA_2_GeI_4_ converts to a monoclinic phase whereas no evidence of phase transition has been detected by lowering temperature (see differential scanning calorimetry measurement of Figure S15). These data could suggest the presence of STEs induced by lattice deformations as a key characteristic of DA_2_GeI_4_, with broad emission that can switched on by low-temperature and high-pressure. Further experimental and computational work will be carried out to get a solid microscopic description of the STEs nature in DA_2_GeI_4_ but it is clear that the present results open a new avenue to search broad – and possibly white – emitters in lead-free layered perovskites.

### Conclusion

We investigated the structural stability and optical properties of DA_2_PbI_4_ and DA_2_GeI_4_ with the aim to provide the evidence of pressure-induced phenomena in 2D perovskites characterized by a long alkyl chain and highlight the role of a different central atom. In this work we also reported a novel lead-free 2D perovskite, namely DA_2_GeI_4_, and we afforded the first high-pressure study of a layered Ge-containing phase.

From a structural point of view, both perovskites present, at ambient pressure, an orthorhombic crystal structure which converts to a monoclinic symmetry at relatively low pressure values (<2 GPa). After the phase transition, further increase of pressure for both materials leads to the separation in two monoclinic phases, with one of the two having a distinct shorter *c*-axis. The present data, together with the few reports on pressure response of 2D perovskites, seem to indicate that phase separation might be a quite common behavior in these systems, differently from 3D perovskites, possibly because of the soft organic spacer which disorders and even melts by increasing pressure. The most remarkable difference between the two samples has been found in the evolution of the optical properties as a function of applied pressure. DA_2_PbI_4_ shows a progressive red shift of the absorption from 2.28 eV at ambient conditions, to 1.64 eV at 11.5 GPa (maximum pressure studied). PL emission is narrow and clearly composed by two components, with the second one appearing in concomitance with the phase separation, significantly shifted to lower energy. Maximum PL intensity if found around 3 GPa. Both structural and optical responses as a function of pressure are different with respect to BA_2_PbI_4_ (the other only alkyl-chain containing 2D perovskite studied to date) suggesting that a systematic study of the role of chain length on these lead-based 2D perovskites is worthy to elucidate the structure-property correlation as a function of pressure and number of carbon atoms of the spacer.

DA_2_GeI_4_ optical properties under pressure revealed a transition from a non-PL system at ambient pressure to a clear broadband emitter, with a large stoke shift, with an intensity maximum at about 3.7 GPa. Absorption measurements shows a first sudden blue-shift (at the orthorhombic to monoclinic phase transition) followed by a continuous red shift. Wide emission with FWHM around 170 nm at about 730 nm in a 2D perovskite containing germanium has not reported previously in the current literature. Although we already determined on other systems that there is an increased octahedral distortion induced by Ge, such evidence of wide emission by a moderate pressure in a lead-free 2D perovskite is a result which deserves deep further investigation. By the appropriate choice of an organic spacer, capable of mimicking through chemical pressure a similar distortion degree, it will be possible to design efficient wide or even white lead-free emitters.

To conclude, our study provided original insight into the role of alkyl chain length on the pressure-induced effects in lead-based 2D perovskites and unveiled a peculiar behavior on Ge-based systems, particularly in the emergence of broadband emission. It seems important to further extend the scope of the present study to both lead-based systems to clarify the role of chain length on the structural and optical properties change with pressure and, even more importantly, to analogous Ge-containing 2D perovskites to understand the extent and origin of broadband emission in order to be able to design suitable materials at ambient condition through chemical pressure effect.

### Limitations of the study

In this study we could not reach a pressure where the samples become fully amorphous, which is most probably located above 20 GPa. The structures above the phase transitions have been refined by the Le Bail method, thus not allowing to provide a deeper knowledge on the structural parameters of the monoclinic phases. Further measurements with different excitonic wavelengths could allow the collection of the Raman spectra of the samples because the PL was recorded in the actual experimental set-up. Finally, the absence of clear contributions by the two phases to the absorbance data of DA_2_PbI_4_ may come from a low amount of the secondary phase and/or by the decreased sensitivity in the high-absorbance region beyond the transition. A definite answer on the relative importance of these two effects may require further study.

## STAR★Methods

### Key resources table

**Table:** 

REAGENT or RESOURCE	SOURCE	IDENTIFIER
**Chemicals, peptides, and recombinant proteins**		

Lead (II) acetate trihydrate, ≥99.99%	Sigma-Aldrich	CAS: 6080-53-4
Germanium dioxide, 99.999%	Acros Organics	CAS: 1310-53-8
Decylamine, 95%	Sigma-Aldrich	CAS: 2016-57-1
Hydriodic acid, 57% w/w aq. soln., stab with 1.5% hypophosphorous acid	Alfa Aesar	CAS: 10,034-85-2
Hypophosphorous acid, 50% w/w aq. soln	Alfa Aesar	CAS: 6303-21-5

**Software and algorithms**		

OriginPro 8.1	Origin Lab Corporation	https://www.originlab.com/
GSAS-II	J. Appl. Cryst. (2013). 46, 544-549	https://subversion.xray.aps.anl.gov/trac/pyGSAS

**Other**		

High-pressure X-ray Diffraction	Elettra Sincrotrone Trieste	https://www.elettra.trieste.it/it/index.html
high-pressure absorbance and PL measurements	Bruker Vertex spectrometer	https://www.bruker.com/en/products-and-solutions/infrared-and-raman/ft-ir-research-spectrometers/vertex-research-ft-ir-spectrometer/vertex-70v-ft-ir-spectrometer.html
Low-temperature PL measurements	Janis cryostat	https://www.lakeshore.com/products/categories/overview/janis-products/cryostats/supervaritemp-cryostat-systems

### Resource availability

#### Lead contact

Further information and requests for resource and reagents should be directed to and will be fulfilled the by Lead Contact, Lorenzo Malavasi (lorenzo.malavasi@unipv.it).

#### Materials availability

This study did not generate unique reagents.

### Experimental model and subject details

Our study does not use experimental models typical in the life sciences.

### Method details

#### Synthesis of DA_2_GeI_4_

DA_2_GeI_4_: all reagents were purchased by Merck Company and used as received. DA_2_GeI_4_ powder was prepared in inert atmosphere under N_2_ flux by a solution method. The followed procedure consisted in the dissolution of a proper amount of GeO_2_ powder in a large excess of both 57% w/w aqueous HI and 50% w/w aqueous H_3_PO_2_, the latter introduced to reduce Ge (IV) and stabilize the reduced oxidation state of Ge. The solution was gradually heated in an oil bath to 100°C under continuous stirring and nitrogen atmosphere to prevent Ge oxidation. After the solid dissolution, a stoichiometric amount of the liquid decylamine was added dropwise. Subsequently, the reaction mixture was cooled down to room temperature obtaining the formation of an orange powder. The precipitate was immediately filtered, dried under vacuum overnight at 65°C and lastly stored in the glove box under Ar atmosphere.

#### Synthesis of DA_2_PbI_4_

DA_2_PbI_4_: for the DA_2_PbI_4_ powder the synthetic procedure also consisted in a solution method but performed, in this case, under ambient conditions. A proper amount of lead (II) acetate was dissolved in a large excess of 57% w/w aqueous HI under magnetic stirring. The solution was heated to 100°C in an oil bath until the solid dissolution, and a stoichiometric amount of the amine was introduced dropwise. Then, a bright orange powder was obtained after cooling down to room temperature. The product was finally filtered and dried at 65°C under vacuum overnight.

#### Characterization of DA_2_GeI_4_ and DA_2_PbI_4_

High-pressure x-ray-diffraction studies were carried out on two samples at the Xpress beamline of Elettra (Lotti et al., 2020). For the pressure run, a membrane-driven symmetric DAC was used together with a PACE5000-based automatic membrane drive. Silicone oil was used as the pressure-transmitting medium (PTM). Pressure was monitored *in situ* by ruby fluorescence method, by including one or more ruby chips (∼10 μm) along with the sample in the pressure cell. Diffraction data collection was performed by a monochromatic circular beam with a wavelength of 0.495 Å and a beam cross-sectional diameter ∼40 μm.

Uncorrected emission spectra were obtained on two samples with an Edinburgh FLS980 spectrometer equipped with a Peltier-cooled Hamamatsu R928 photomultiplier tube (185 nm–850 nm). An Edinburgh Xe900 450 W Xenon arc lamp was used as exciting light source. Corrected spectra were obtained *via* a calibration curve supplied with the instrument.

To record the 77 K luminescence spectra on one sample (DA_2_GeI_4_), the sample was put in glass tubes (2 mm diameter) and inserted in a special quartz Dewar, filled up with liquid nitrogen. For solid samples, λem have been calculated by corrected emission spectra obtained from an apparatus consisting of a barium sulfate coated integrating sphere (4 or 6 inches), a 450W Xe lamp (λ_exc_ = tunable by a monochromator supplied with the instrument) as light sources, and a R928 photomultiplier tube as signal detectors, following the procedure described by DeMello et al. (deMello et al., 1997). Experimental uncertainties are estimated to be ± 20% for emission quantum yields, ± 2 nm and ± 5 nm for emission peaks, respectively.

For the high-pressure absorbance and PL measurements two samples, DA_2_GeI_4_ and DA_2_PbI_4_, were prepared in DACs utilizing 400 μm culets. A 250 μm thick Re gasket was pre-indented to ∼45 μm, and a ∼200 μm hole was laser-drilled for the sample chamber. The perovskite samples were loaded as ∼10 μm thick pressed pellets within an inert Ar glove box (MBraun, <0.5 ppm O_2_/H_2_O). After sealing under ∼1 atm Ar, the samples were loaded with high-pressure Ar gas at ∼1 kbar, which served as the pressure transmitting medium and absorbance reference. A ruby chip was used for pressure calibration. Optical absorbance measurements were performed on two samples on using a Bruker Vertex spectrometer with Hyperion microscope utilizing two-sided, knife-edge collimation (∼50 μm × 50μm) and reflecting objectives. A high-intensity water-cooled tungsten lamp served as the NIR-VIS light source. PL measurements were performed using a 532 nm laser excitation source with emission collected in the backscatter geometry through a 50 μm confocal filter and focused onto the entrance slit of a Princeton Instruments spectrograph (SP2750) utilizing a 300 gr/mm grating (500 nm blaze) and liquid-nitrogen-cooled CCD detector. For the DA_2_GeI_4_ sample, a 405 nm laser excitation source was also tested, but no PL was detected at room temperature.

Low temperature photoluminescence (PL) measurements were performed on two samples keeping the sample powders inside a flowing liquid nitrogen cryostat (Janis SVT-200-05) equipped with a Lakeshore 331 temperature controller. The sample was excited with a continuous wave laser (Spectra-Physics Millennia IV) operating at 533nm with 30 mW/cm^2^ excitation power density. PL signal was collected with achromatic lenses, spectrally dispersed with a grating spectrograph (Acton SpectraPro 2300i) and finally detected using an Hamamatsu digital camera (model C4742-95).

### Quantification and statistical analysis

Our study does not include statistical analysis or quantification.

## Data Availability

• All data reported in this paper will be shared by the lead contact upon request. • This paper does not report original code. • Any additional information required to reanalyze the data reported in this paper is available from the lead contact upon request.
