# Supplementary material for: Pressure response of decylammonium-containing 2D iodide perovskites
Source: iScience. 2022 Mar 11;25(4):104057. doi: 10.1016/j.isci.2022.104057 (PMC8957026; doi:10.1016/j.isci.2022.104057)
Supplement: Document S1. Figures S1–S14 [file mmc1.pdf]

iScience, Volume *25*

Supplemental information

Pressure response of decylammonium-containing 2D iodide perovskites

Marta Morana, Rossella Chiara, Boby Joseph, Thomas B. Shiell, Timothy A. Strobel, Mauro Coduri, Gianluca Accorsi, Ausonio Tuissi, Angelica Simbula, Federico Pitzalis, Andrea Mura, Giovanni Bongiovanni, and Lorenzo Malavasi

SUPPLEMENTAL INFORMATION

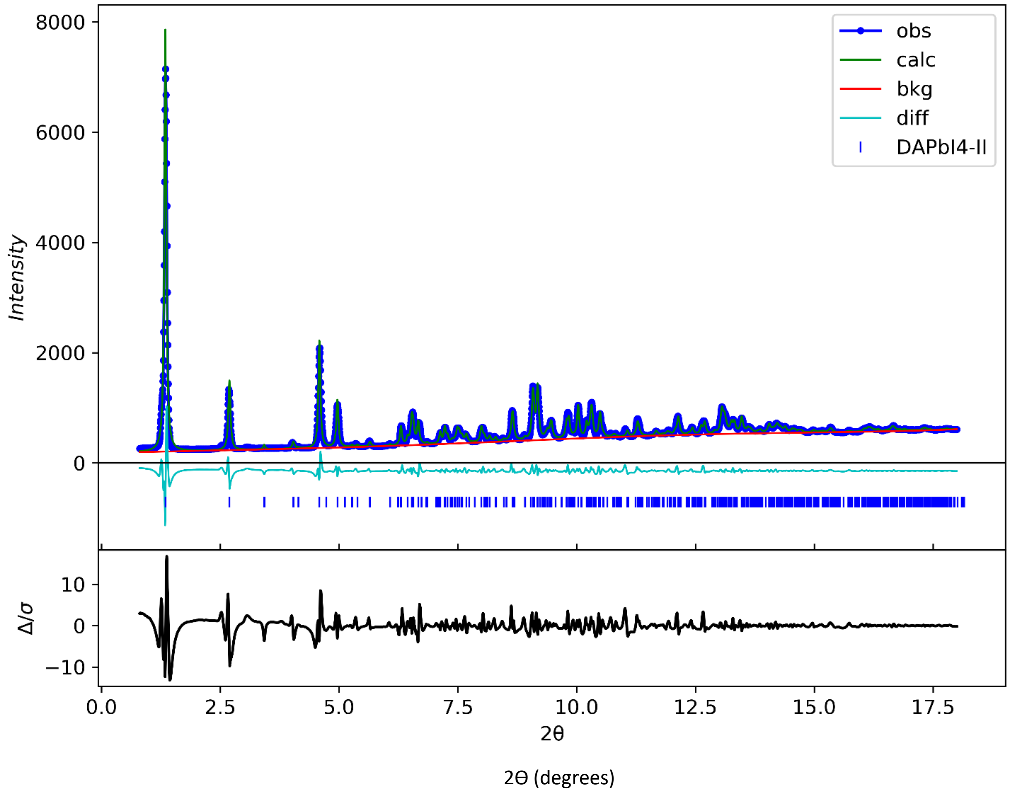

Figure S1. Refinement of DA_2PbI_4 pattern. Related to Figure 1. Refined ambient pressure pattern of DA_2PbI_4 (λ=0.495Å) in S.G. *Pbca*

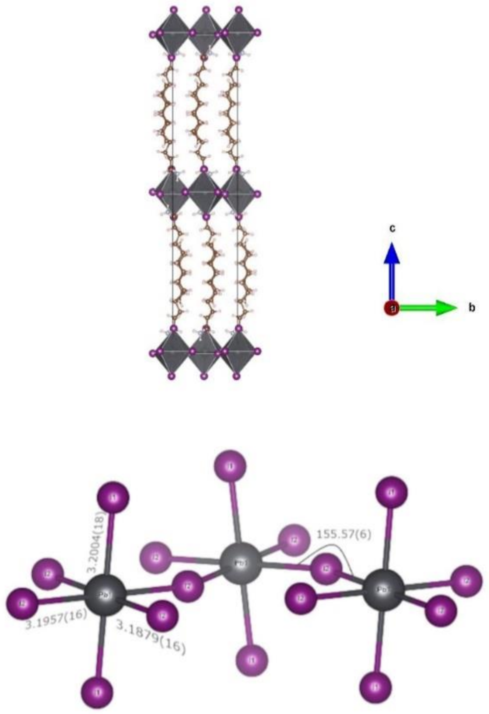

Figure S2. DA_2PbI_4 structure. Related to Figure 1. Sketch of the crystal structure and bond angles and lengths of DA_2PbI_4.

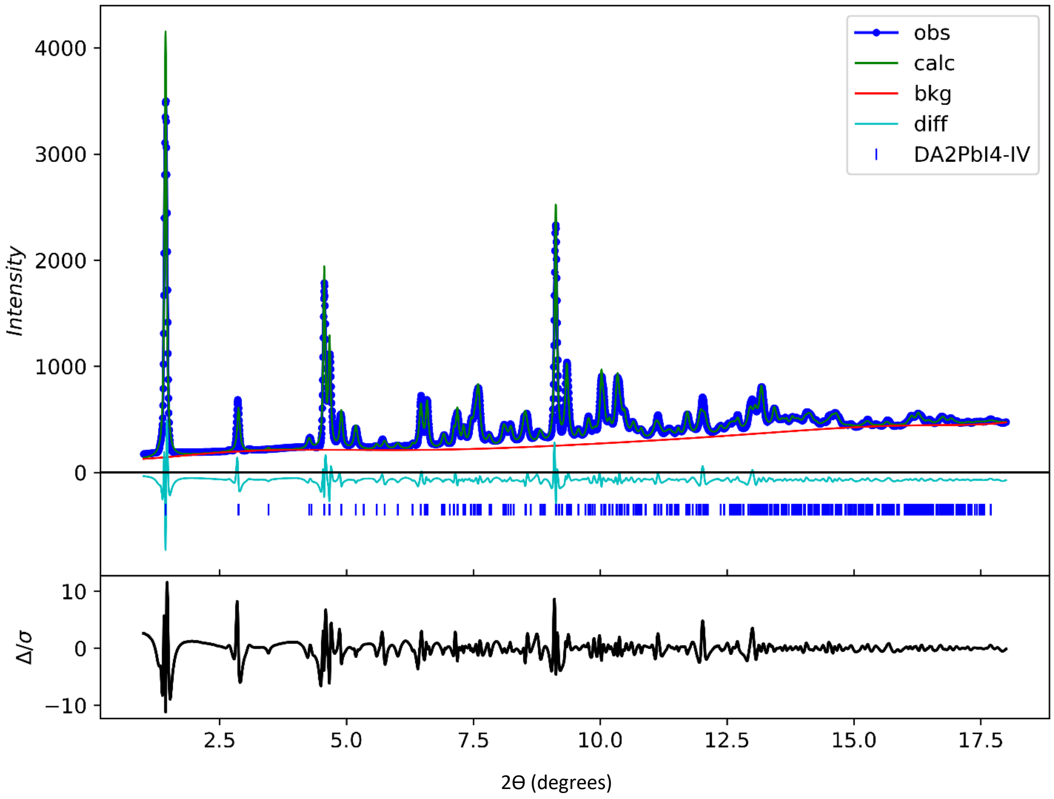

Figure S3 = Refinement of DA_2PbI_4 pattern. Related to Figure 1. Refined pattern of DA_2PbI_4 (λ=0.495Å) at 0.36 GPa in S.G. P2_1/*a*

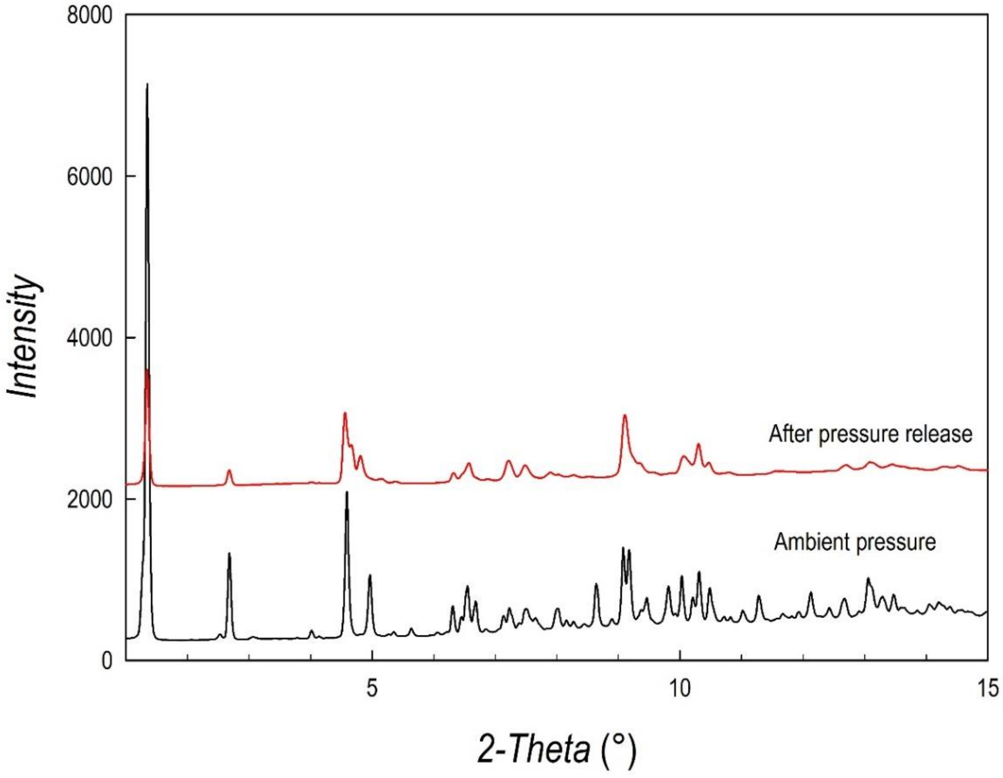

Figure S4 = Refinement of DA_2PbI_4 pattern. Related to Figure 1. XRD pattern of DA_2PbI_4 at ambient pressure (black line) and after pressure release (red line)

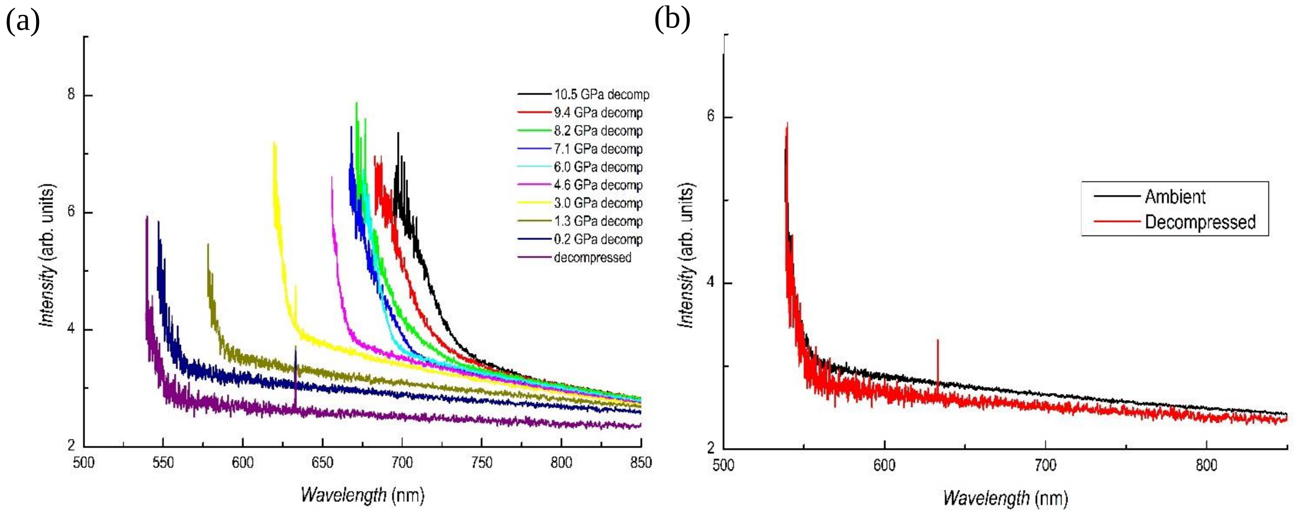

Figure S5. DA_2PbI_4 spectra during decompression. Related to Figure 3. (a) absorption spectra as a function of pressure for DA_2PbI_4 during decompression; (b) ambient pressure spectra before applying pressure (black line) and after pressure release (red line)

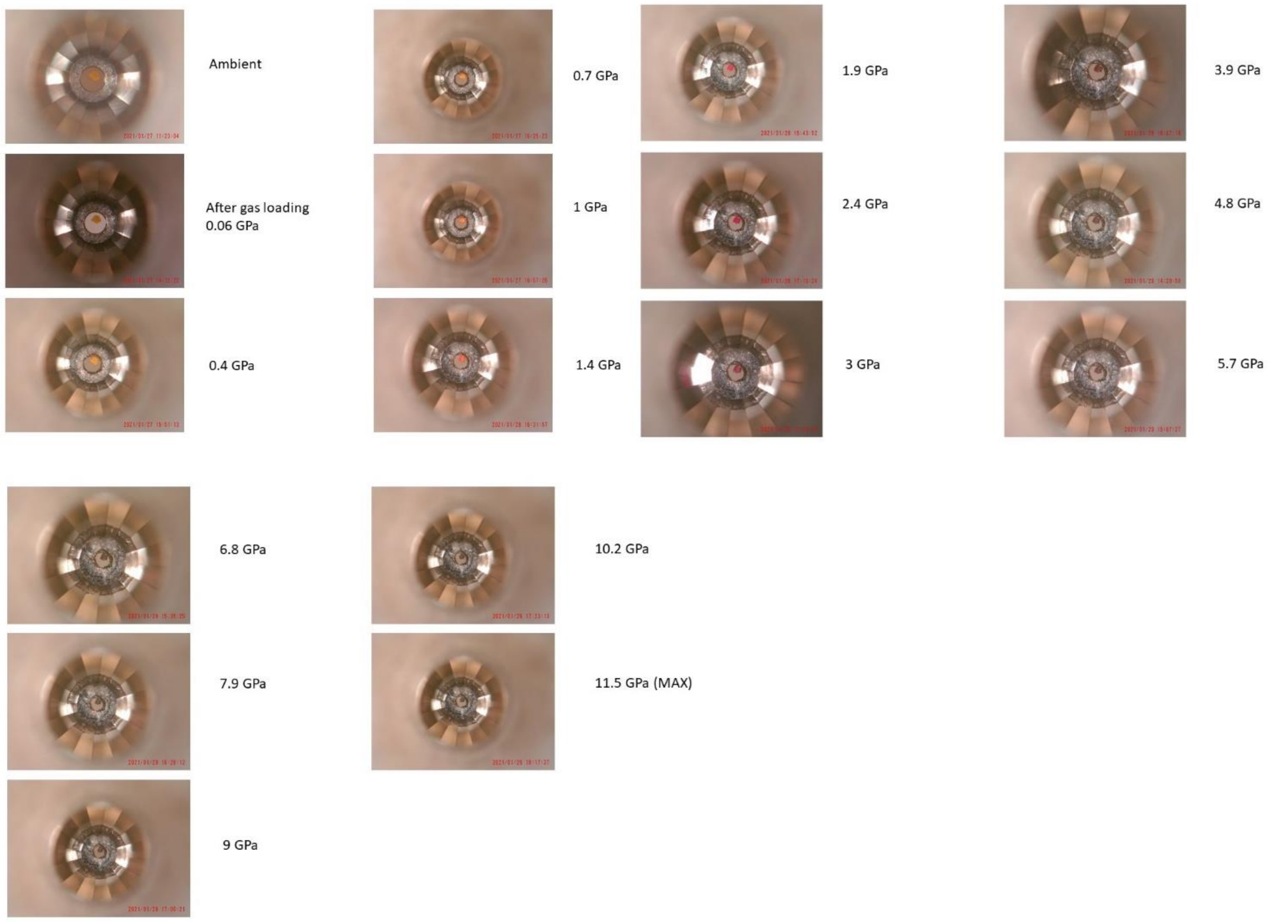

Figure S6. **Images of DA_2PbI_4.** Related to Figure 4. Optical micrographs of DA_2PbI_4 during compression.

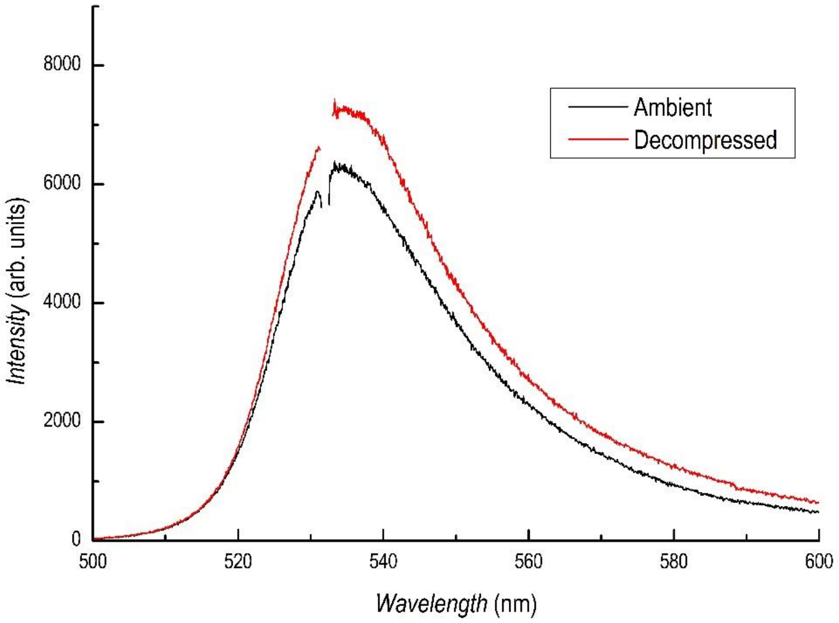

Figure S7. DA_2PbI_4 PL during decompression. Related to Figure 4. PL of DA_2PbI_4 at ambient pressure before applying pressure (black line) and after pressure release (red line). Missed part are due to the removal of the laser contribution.

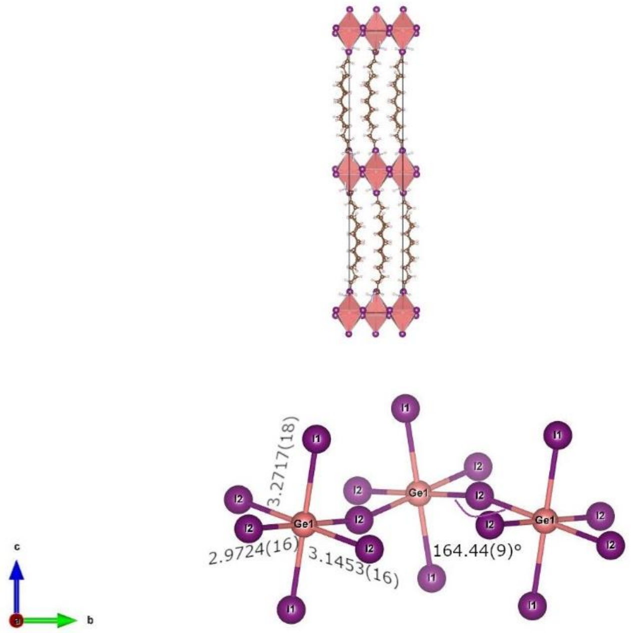

Figure S8. DA_2GeI_4 structure. Related to Figure 5. Sketch of the crystal structure and bond angles and lengths of DA_2GeI_4.

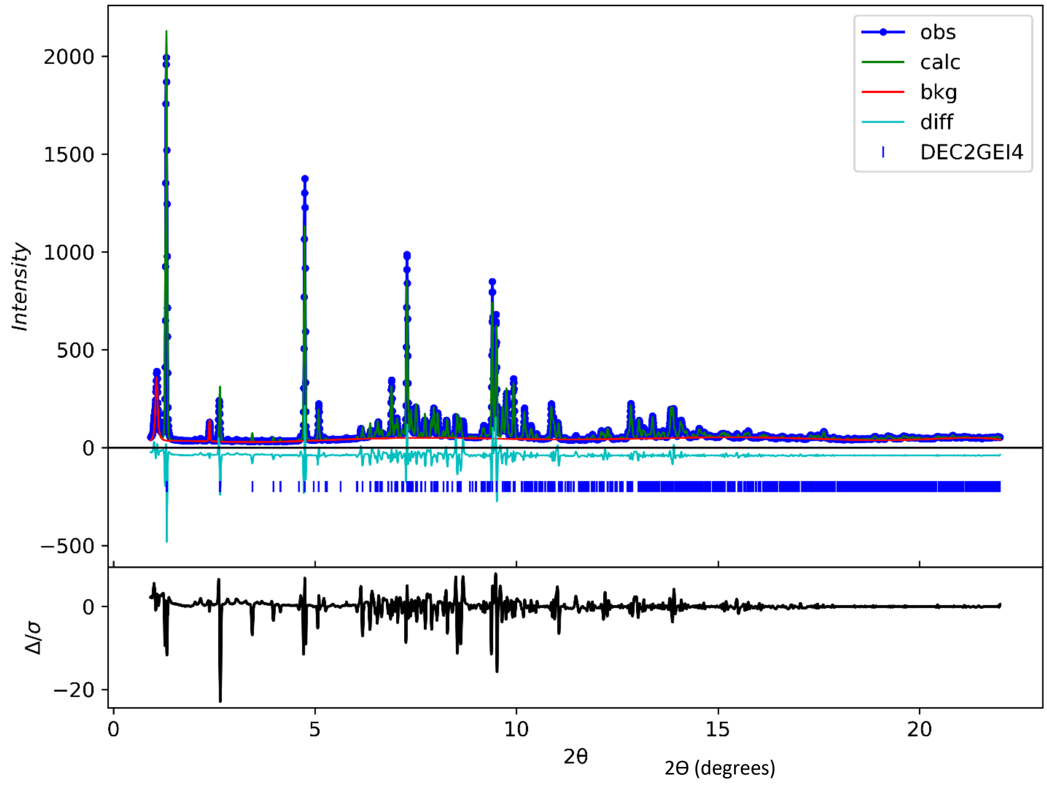

Figure S9. Refinement of DA_2GeI_4 pattern. Related to Figure 5. Refined ambient pressure pattern of DA_2GeI_4 (λ=0.495Å) in S.G. *Pbca*

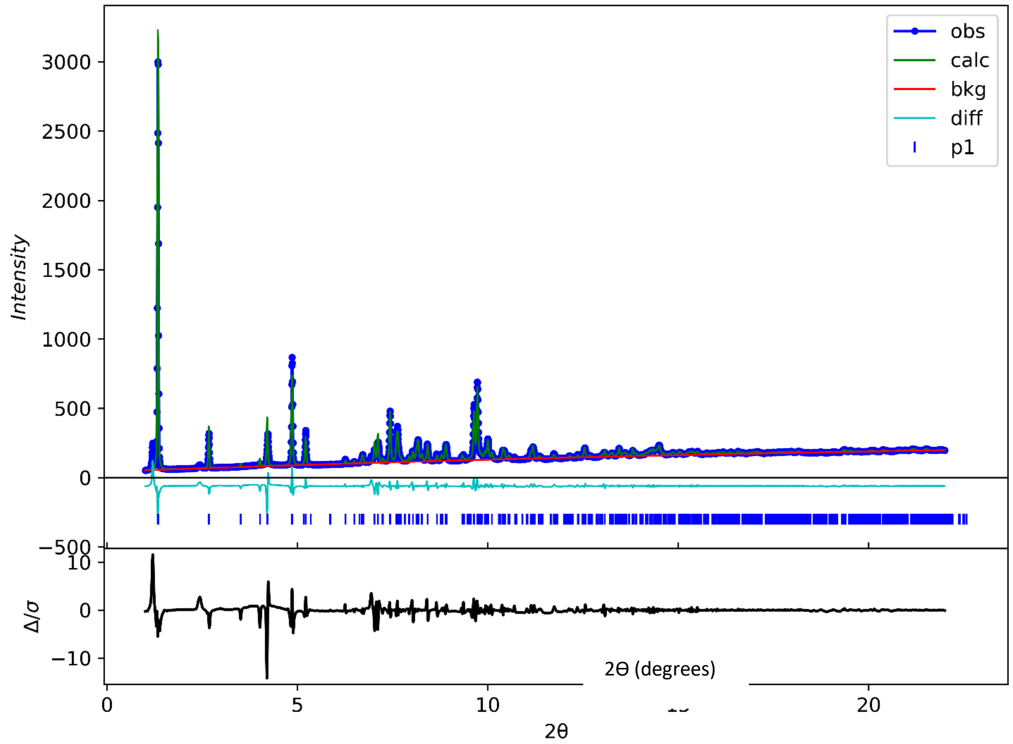

Figure S10. Refinement of DA_2GeI_4 pattern. Related to Figure 5. Refined pattern of DA_2GeI_4 (λ=0.495Å) at 0.56 GPa in S.G. $P2_1/a$

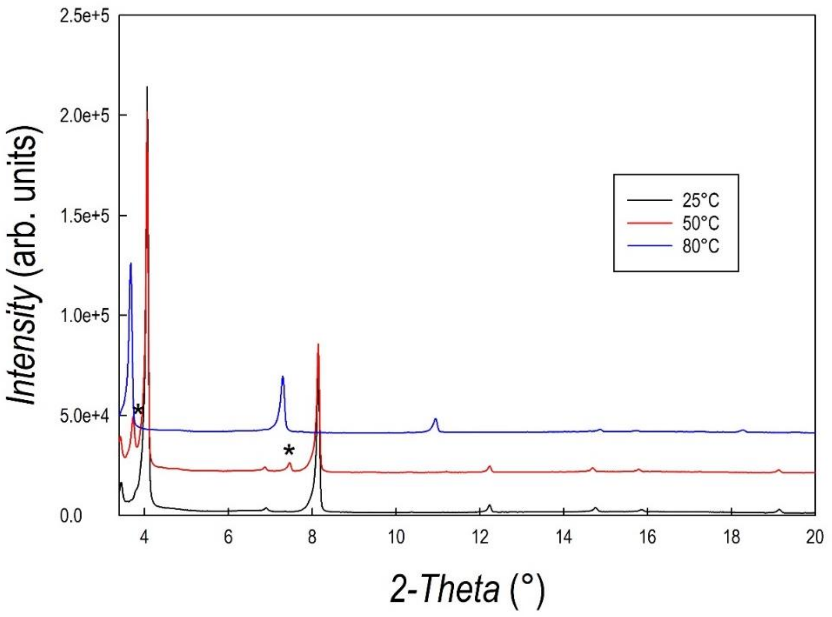

Figure S11. Variable-temperature patterns of DA_2GeI_4. Related to Figure 8. XRD pattern of DA_2GeI_4 as a function of temperature. Asterisks mark the second peak in the two phases system.

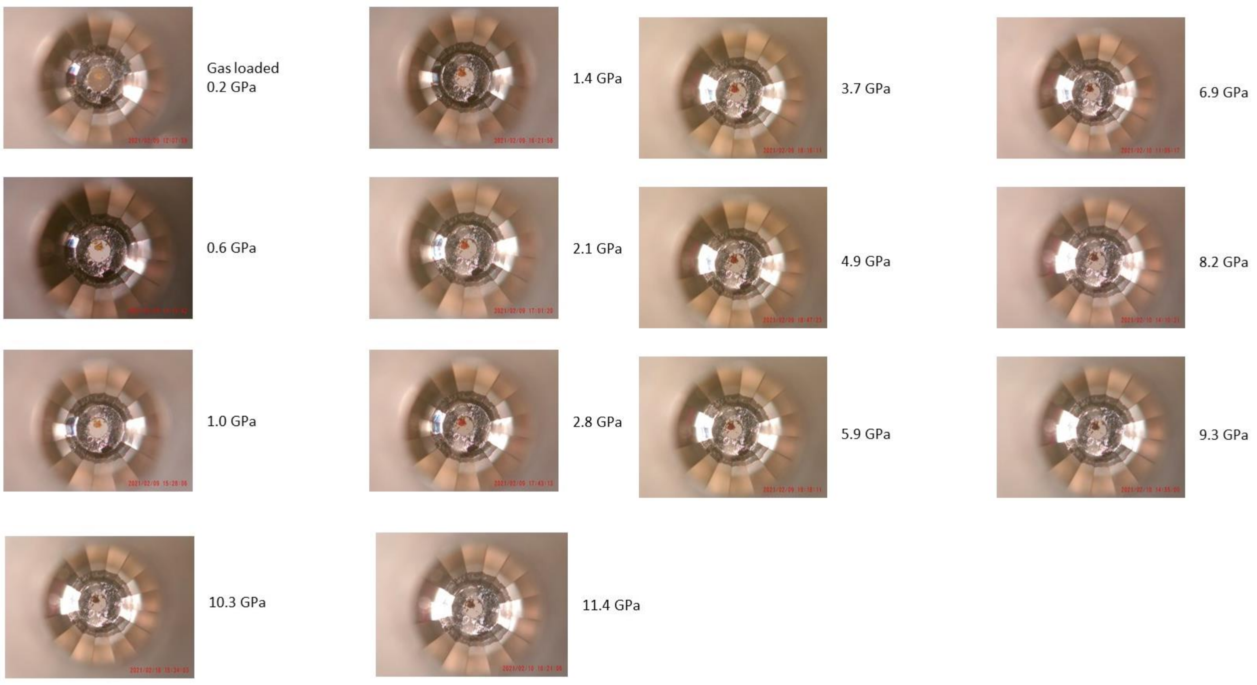

Figure S12. Images of DA_2GebI_4. Related to Figure 8. Optical micrographs of DA_2GeI_4 during compression.

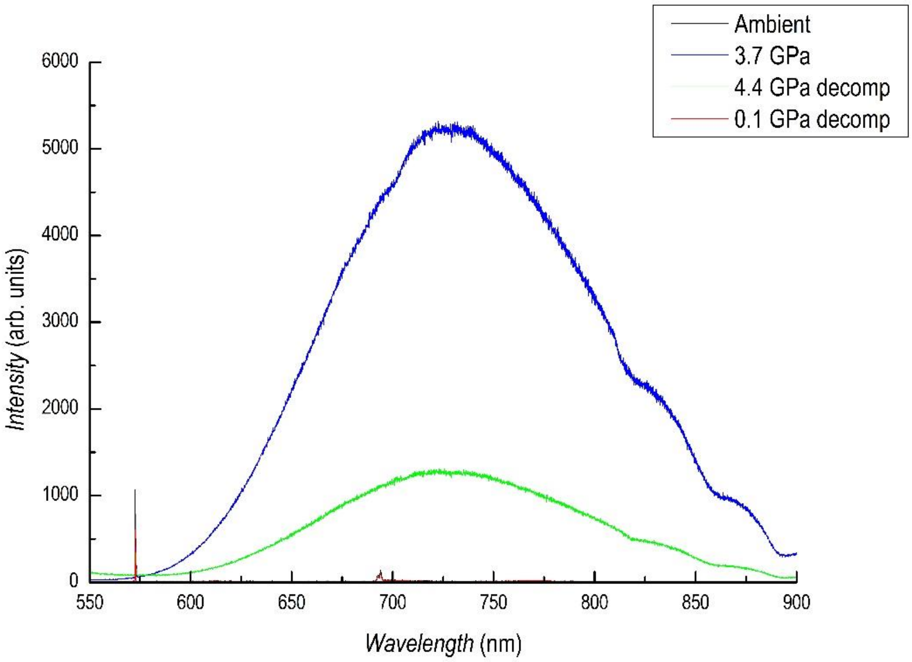

Figure S13. PL data during decompression of DA_2GeI_4. Related to Figure 8. PL of DA_2GeI_4 at ambient pressure before applying pressure (black line) and after pressure release (red line) and at 3.7 GPa during compression (blue line) and at 4.4 GPa during decompression (red line). See main text for details. Signal around 57’ nm comes from the diamond while small peaks around 680 comes from the ruby signal.

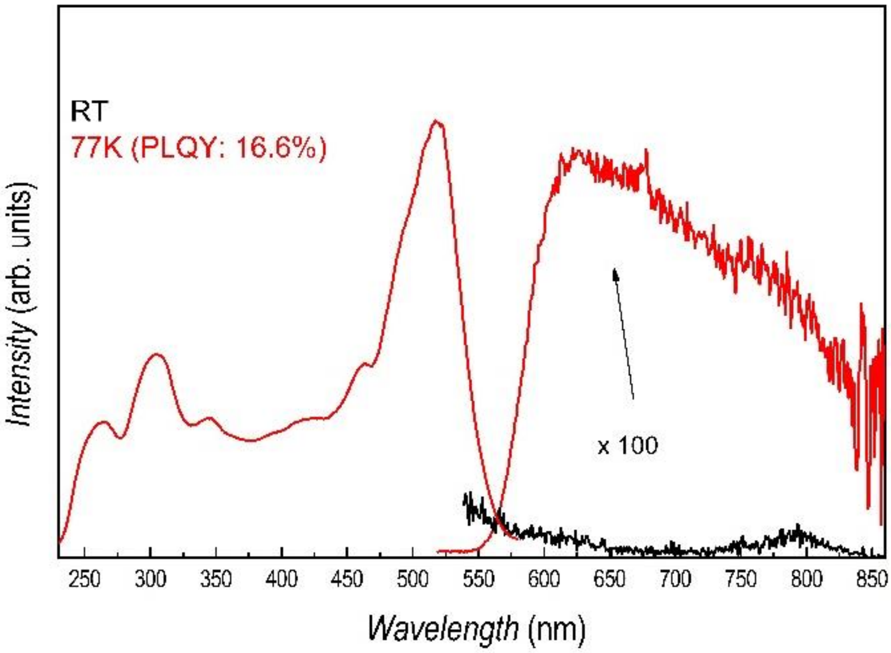

Figure S14. PL data at low-T of DA_2GeI_4. Related to Figure 8. PL data at room temperature (black line) and 77 K (red line) for DA_2GeI_4.

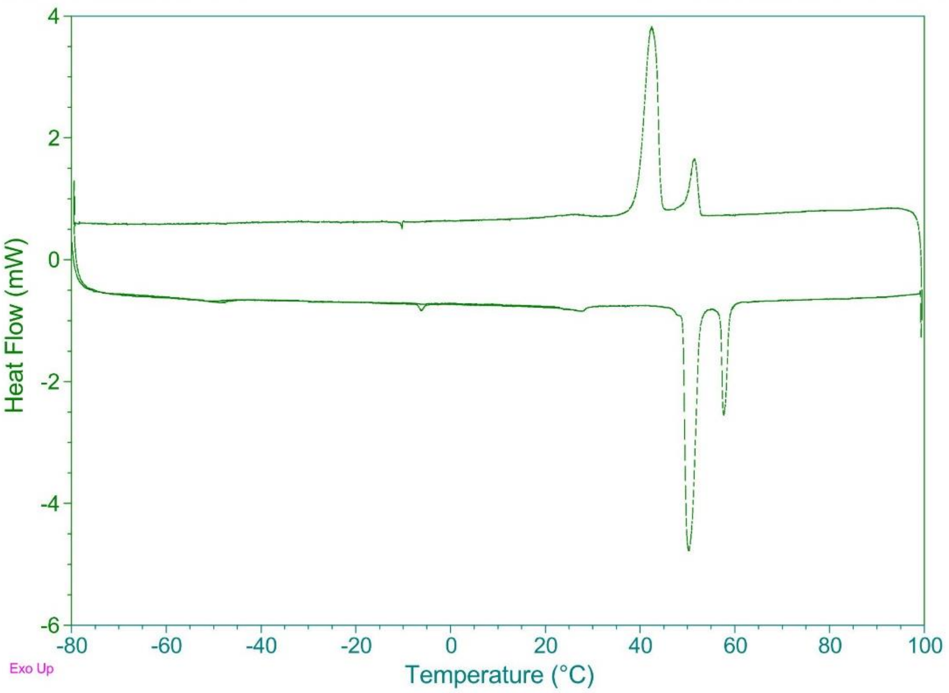

Figure S15. Thermal analysis of **DA_2GeI_4**. Related to Figure 8. DSC traces of for DA_2GeI_4 (exo up)
